# A novel tumor-targeting strain of *Xenorhabdus stockiae* exhibits potent biological activities

**DOI:** 10.3389/fbioe.2022.984197

**Published:** 2022-09-07

**Authors:** Chao Zhang, Hanna Chen, Stephan Hüttel, Shengbiao Hu, Wangyue Zhang, Xuezhi Ding, Jia Yin, Yulong Yin, Rolf Müller, Liqiu Xia, Youming Zhang, Qiang Tu

**Affiliations:** ^1^ Hunan Provincial Key Laboratory of Microbial Molecular Biology, State Key Laboratory of Developmental Biology of Freshwater Fish, College of Life Science, Hunan Normal University, Changsha, China; ^2^ Helmholtz International Lab for Anti-infectives, Shandong University–Helmholtz Institute of Biotechnology, State Key Laboratory of Microbial Technology, Shandong University, Qingdao, China; ^3^ Helmholtz Institute for Pharmaceutical Research, Helmholtz Centre for Infection Research and Department of Pharmacy Biotechnology, Saarland University, Saarbrücken, Germany; ^4^ CAS Key Laboratory of Quantitative Engineering Biology, Shenzhen Institute of Synthetic Biology, Shenzhen Institute of Advanced Technology, Chinese Academy of Sciences, Shenzhen, China

**Keywords:** Xenorhabdus stockiae, HN_xs01, biological activities, tumortargeting, entomopathogenic nematodes

## Abstract

*Xenorhabdus* are symbionts of soil entomopathogenic nematodes of the genus *Steinernema* presenting two distinct forms in their life cycle, and can produce a broad range of bioactive compounds. In this study, a novel *Xenorhabdus stockiae* strain HN_xs01 was isolated from a soil sample via an entrapment method using *Galleria melonella* nematodes. The supernatants of strain HN_xs01 exhibited antimicrobial properties against Gram-negative and Gram-positive bacteria, and insecticidal properties against *Helicoverpa armigera* larvae, and antitumor properties as well. Moreover, three linear rhabdopeptides (**1**, **2** and **3**) were identified from strain HN_xs01 using nuclear magnetic resonance analysis, which exhibited significant cytotoxic activity against the human epithelial carcinoma cell line A431 and the human chronic myelogenous leukemia cell line K562. Some bacteria have been reported to colonize the tumor region, and we determined that HN_xs01 could grow in tumor xenografts in this study. HN_xs01 invaded and replicated in B16 melanoma cells grafted into C57BL/6 mice, resulting in tumor inhibition. Moreover, strain HN_xs01 not only merely aggregated in the tumor environment, but also prevented pulmonary metastasis. It caused fragmentation of vessels and cell apoptosis, which contributed to its antitumor effect. In conclusion, *X. stockiae* HN_xs01 is a novel tumor-targeting strain with potential applications in medicinal and agricultural fields.

## Introduction

The genus *Xenorhabdus* is a mutualistic symbiont of entomopathogenic nematodes (EPNs) of the genus *Steinernema*. *Xenorhabdus spp.* are a motile, Gram-negative, entomopathogenic bacteria that present two distinct forms, primary and secondary form, in their life cycle. The primary form of *Xenorhabdus* is retained in the intestine of infective stage nematodes, contributes to faster and greater reproduction of nematodes, and is less stable than the secondary form, which is significant for the primary form in the monoxenic culture of nematodes *in vitro* (Thomas and George, 1979; Akhurst, 1980). The infective juveniles invade insect larvae through natural openings and release bacteria from their intestine to the host’s hemocoel, resulting in the production of insecticidal proteins and inhibitors by overcoming the insect immune system and countering the effect of various antibiotics by inhibiting other microorganisms. Then, the bacteria multiply rapidly and provide nutrients for the reproduction of the nematodes (Sicard et al., 2006; Singh et al., 2015). Subsequently, nematode progeny associated with *Xenorhabdus* emerge from the cadaver to seek a new host.

Multiple compounds from various *Xenorhabdus* strains have been isolated and identified. These compounds, including proteinaceous and non-proteinaceous compounds, have diverse chemical structures and a wide range of bioactivities, such as antibacterial, antifungal, insecticidal, nematicidal, and cytotoxic properties (Mcinerney et al., 1991; Samaliev et al., 2000; Brown et al., 2006; Gualtieri et al., 2009; Qin et al., 2013; Fang et al., 2014). In addition, multiple secondary metabolites produced by *Xenorhabdus* are mostly synthesized by multienzyme thiotemplate mechanisms, such as nonribosomal peptide synthetases (NRPSs) and polyketide synthases (PKSs) (Staunton and Weissman, 2001; Strieker et al., 2010). Rhabdopeptides biosynthesized by NRPSs are very common in *Xenorhabdus* species, and show cytotoxic and antiprotozoal activities that might be involved in the protection of the insect cadaver (Bozhuyuk et al., 2016; Cai et al., 2017; Bi et al., 2018). Several *Xenorhabdus* species have been revealed to contain about twenty biosynthetic gene clusters, most of which are uncharacterized (Chaston et al., 2011; Cimen et al., 2022). Therefore, *Xenorhabdus spp.* could be a promising source of natural products.

The application of bacteria in cancer therapy has been a research focus for many years. *Cancer* therapy studies over the last 2 decades have revealed that several genera of bacteria, such as *Clostridium novyi*-NT, non-pathogenic *Salmonella typhimurium*, *Escherichia coli* Nissle 1917, and *Bifidobacterium*, can efficiently colonize and proliferate in certain tumors, resulting in the suppression of tumor growth (Tang et al., 2009; Yu et al., 2012; Zhang et al., 2012; Roberts et al., 2014). The mechanisms by which these bacteria can target different intratumoral regions include chemotaxis, preferential growth, and hypoxic germination. Obligate anaerobes including *Clostridium* and *Bifidobacterium* do not survive in oxygenated regions but are highly effective at accumulating in large hypoxic regions of tumors (Agrawal et al., 2004; Tang et al., 2009). Facultative anaerobes such as *Salmonella* and *E. coli* Nissle 1917 utilize chemotaxis to migrate towards compounds produced by tumors, leading to their preferential growth of these bacteria in tumor-specific microenvironments (Jean et al., 2008). Genetically modified strains coupled with antitumor substances, DNA fragments, or enzymatic drug activation are rapidly being developed to augment the efficacy of bacteria in antitumor therapy (Jean et al., 2008). Moreover, the development of new bacteria for cancer therapy is an urgent strategy.

*X. stockiae* is a symbiotic partner of *Steinernema siamkayai*, first described in 2006 (Tailliez et al., 2006). In this study, the bacterial strain HN_xs01, identified as *X. stockiae*, was isolated from soil samples. The anti-tumor effect of HN_xs01 exhibiting potent bioactivities on the mice bearing B16 mouse melanoma have been investigated *in vivo*. The strain HN_xs01 efficiently colonized the tumor region and suppressed the growth of tumors via intravenous and intratumoral injection, which laid an important foundation for development of *Xenorhabdus* species for cancer therapy.

## Materials and methods

### Bacterial strain isolation

*Xenorhabdus* strains were isolated from nematodes obtained from the foot of Yuelu Mountain (City of Changsha, Hunan Province, China) by an entrapment method using *Galleria melonella* (Bedding and Akhurst, 1975). Adult instars of *G. melonella* were placed in 500 cm^3^ soil and incubated until death. The dead *G. melonella* was removed and washed with sterile water. Haemolymph samples were collected, plated onto NBTA medium (4.5 g nutrient agar, 0.004 g triphenyltetrazolium chloride (TTC), and 0.0025 g bromothymol blue per 100 ml distilled water), and incubated at 30 ^o^C for 48 h. To obtain pure cultures, blue colonies were streaked on LB plates (1 g peptone, 0.5 g yeast extract, and 1 g NaCl per 100 ml distilled water) and incubated at 30 ^o^C for 36 h.

### Phylogenetic analysis

Genomic DNA was extracted using a genomic DNA isolation kit (TIANGEN BIOTECH). PCR products were gel-purified using a PCR purification kit (TIANGEN BIOTECH) and sequenced using Invitrogen (Shanghai). The *16S* rRNA gene sequence of strain HN_xs01 was aligned using SINA sequence alignment software. The aligned sequences were imported into the database of the Living Tree Project release 121 database using the ARB software package (Yarza et al., 2008). The phylogenetic tree was reconstructed using the neighbor-joining method with Jukes–Cantor correction. For evolutionary phylogenetic analysis, bootstrap analysis was used to evaluate tree topology by performing 1,000 replications.

### Isolation, purification and chemical characterization of cytotoxic compounds

For the detection and purification of cytotoxic compounds from HN_xs01, 5 L fermentation broth was mixed with the adsorbent resin XAD-16 at 30°C, 150 rpm for 4 days. The cells and resin were harvested by centrifugation and extracted using ethyl acetate. The extract was evaporated, redissolved in methanol, and then subjected to C18 column chromatography (Luna RP-C18 column, 100 × 2 mm, 2.5 μm particle size, and pre-column C18, 8 × 3 mm, 5 μm). Collected fractions were tested in a bioassay with CHO-K1 cells, and bioactive fractions were further purified to obtain three compounds via semi-preparative C18 chromatography. The planar structure of these compounds was elucidated using various spectroscopic/spectrometric techniques (see supplementary information), including high-resolution ESI-MS and MS/MS as well as 1D and 2D nuclear magnetic resonance (NMR) experiments (^1^H, HSQC, HMBC, COSY). Cytotoxic activities of the three compounds against the A431 (human epithelial carcinoma) and K562 (human chronic myelogenous leukemia) cell lines were evaluated.

### Animal tumor models

Animal experiments were conducted in accordance with the National Institutes of Health Guide for the Care and Use of Laboratory Animals and approved by the Animal Ethics Committee of Hunan Normal University. Specific-pathogen-free male C57BL/6 mice were purchased from the SLRC Laboratory Animal Company, Hunan Province, China. The C57BL/6 mice, 6–8 weeks old, were injected with 5 × 10^5^ B16 melanoma cells in the right front and left back hypodermic injections. After 7–10 days, when tumor volumes had reached ∼5 mm^3^, the animals were randomly assigned to different groups and observed continuously after injection. After a defined time, the mice were sacrificed by cervical dislocation.

### Tolerance analysis of HN_xs01 in healthy mice after intravenous injection

C57BL/6 mice (two mice per group) were injected intravenously with six concentrations of strain HN_xs01 (1 × 10^6^ CFU/100 μL, 5 × 10^6^ CFU/100 μL, 1 × 10^7^ CFU/100 μL, 5 × 10^7^ CFU/100 μL, 1 × 10^8^ CFU/100 μL, or 5 × 10^8^ CFU/100 μL) or with 100 μL sterile PBS (pH 7.4) for the control group. Survival was observed continuously for 10 days after injection.

### Bacterial distribution and antitumor activity of HN_xs01

C57BL/6 mice bearing B16 tumors were injected intratumorally with HN_xs01 (1 × 10^8^ CFU/100 μL) and 100 μL sterile PBS (control), respectively. After intratumoral injection, tumor volumes and mouse weight were measured for 10 days (seven mice per group). C57BL/6 mice bearing B16 tumors were injected intravenously with HN_xs01 (5 × 10^7^ CFU/100 μL), *E. coli* Nissle 1917 (2 × 10^6^ CFU/100 μL) (Stritzker et al., 2007), and 100 μL sterile PBS (control), respectively. The intravenous injection was performed twice, with the secondary injection administered 6 days after the first injection (four mice per group). The mice were sacrificed after 10 days. Tumors, livers, spleens, and kidneys were excised, weighed, and homogenized. Then serial dilutions were plated on NBTA or LB plates, and afterwards, colonies were counted, and CFU/g tissue was calculated.

### Pulmonary metastasis

Mice bearing B16 tumors received intravenous injections of HN_xs01 (5 × 10^7^ CFU/100 μl) and sterile PBS (control), respectively. After the injection, the mice were weighed daily and sacrificed by cervical dislocation after 7 days. Tumors, hearts, livers, spleens, lungs, and kidneys were isolated, and the tumor nodes were counted. Afterwards, the tissues were minced, serial dilutions were plated on LB agar, and the CFU/g was calculated after 24–48 h.

### Histological analysis

For histological studies, the mice were sacrificed 6 days after intratumoral injection post. Tumors were fixed in 4% paraformaldehyde at room temperature overnight and then stained with hematoxylin and eosin (H&E) to examine tissue morphology and necrosis. The sections were observed and imaged under a microscope.

### Mouse survival rate

C57BL/6 mice bearing B16 tumors (10 mice per group) were administered strain HN_xs01intravenously (5 × 10^7^ CFU/100 μl) and intratumorally (1 × 10^8^ CFU/100 μl) and then observed for 32 days. The control group received an equivalent volume of PBS.

### Statistical analysis

Statistical significance for all experiments was evaluated using the Student’s t-test in the software of GraphPad Prism 5.0 (Xu et al., 2020). If the *p* value was below 0.05, the difference was considered significant.

## Results

### HN_xs01 identified as *X. stockiae* via phylogenetic analysis and its growth characteristics

**FIGURE 1:**
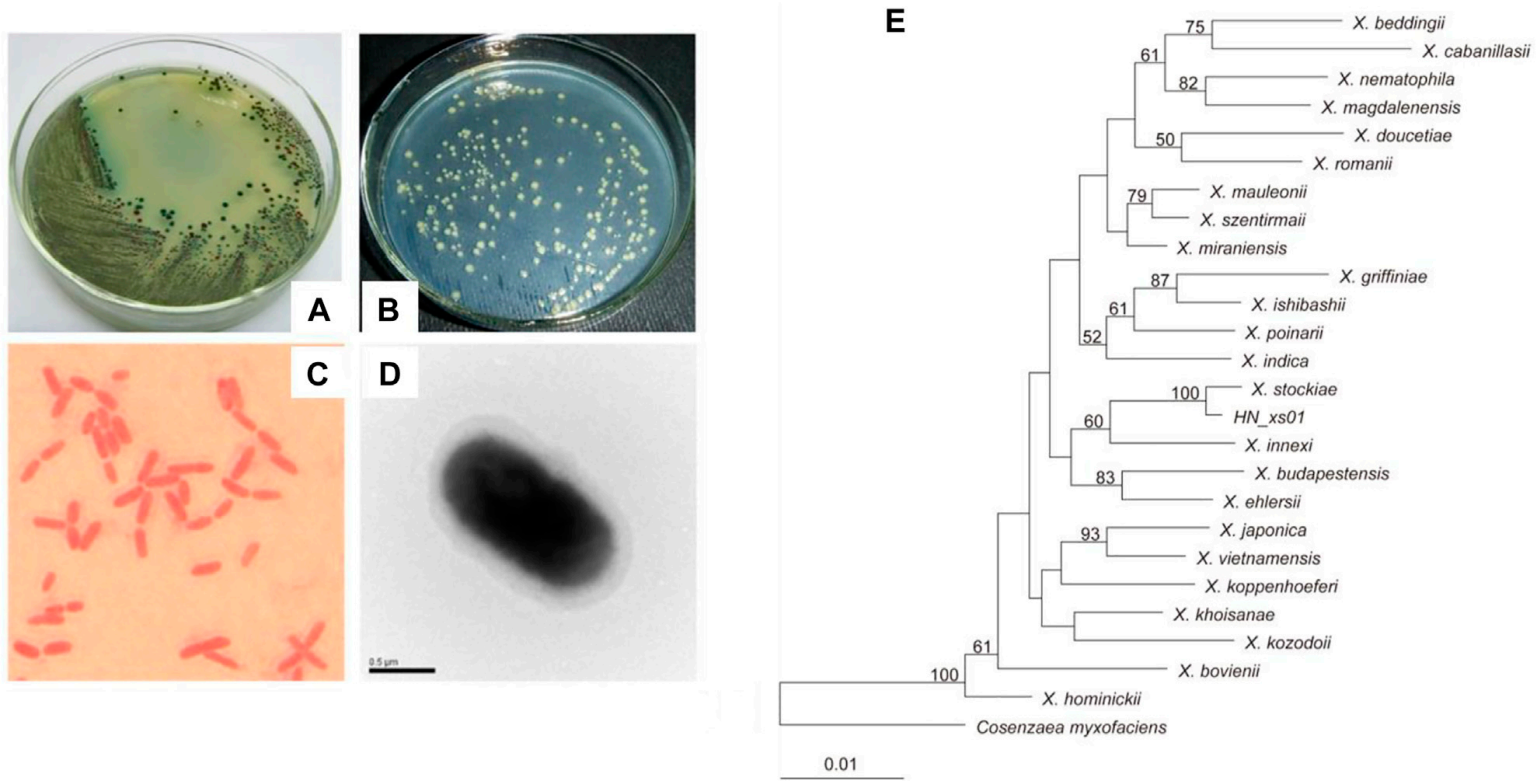
Morphology and phylogenetic analysis of strain HN_xs01. **(A)** Colony morphology of HN_xs01 on NBTA plate. **(B)** Colony morphology of HN_xs01 on LB plate. **(C)** Sarranine staining of HN_xs01. **(D)** The transmission electron microscopy of HN_xs01. **(E)** Phylogenetic dendrogram of HN_xs01. Bootstrap values are based on 1,000 replicates. The dendrogram was obtained by distance matrix analysis of *16S* rRNA gene sequences.

The bacterium HN_xs01 exhibited blue and red colonies on NBTA plates. The blue colonies are the primary form of strain HN_xs01, which can reduce TTC and absorb bromothymol blue. The red colonies are the secondary form of strain HN_xs01, which reduces TTC but does not absorb bromothymol blue and appears as red colonies (Figures 1A,B). HN_xs01 is a rod-shaped Gram-negative bacterium (approximately 1 µm in width; 1–3 µm in length) that does not form spores and is encapsulated by a thick layer, observed using a transmission electron microscopy (Figures 1C,D). The *16S* rRNA gene sequence of strain HN_xs01 was submitted to the GenBank database, and accession number HQ840745 was assigned. Phylogenetic analysis based on *16S* rRNA gene sequences revealed that strain HN_xs01 was 99% similar to *X. stockiae* (Figure 1E), suggesting that they belong to the same species.

Analysis of growth kinetics of the primary and secondary forms of strain HN_xs01 indicated that the growth of the primary form was slower than that of the secondary form after 12 h. Growth of the secondary form began to slow down after 12 h, whereas the primary form continued to grow, resulting in a significantly greater OD_600_ in LB broth and delayed entry into the stationary phase compared with the secondary form (Supplementary Figure S1A). Thus, the primary form has a growth advantage over the secondary form in LB cultures. The pH of the two forms rose sharply between 12 and 21 h and then stabilized (Supplementary Figure S1B). No significant differences were observed in the pH values of the primary and secondary forms during the culture process. The optimal temperature and pH ranges for growth were similar for the two forms. HN_xs01 grew over a wide temperature range, from 18 to 42°C, with an optimal growth temperature of 32°C (Supplementary Figure S1C). Additionally, HN_xs01 grew over a broad pH range from 4.6 to 8.5. The optimal pH for growth of the two forms was 6.6 and 8.2, respectively (Supplementary Figure S1D).

### The supernatants of the primary and secondary forms of the strain HN_xs01 exhibited potent biological activities

Strain HN_xs01 exhibited broad-spectrum antimicrobial activities, including potent antibacterial activities against *S. typhimurium* and *E. coli* (Supplementary Figure S2A). The major antimicrobial compounds were produced after 12 h and reached optimal activity at 24 h. In addition, the primary form showed better antimicrobial activities than the secondary form, as evidenced by the greater zones of inhibition of the primary form. Insecticidal activity was evaluated via the injection of *H. armigera* larvae, and no significant differences were observed between the primary and the secondary forms. Supernatants of 72 h cultures had the highest insecticidal toxicity, and by 6 h after injection, the mortality of *H. armigera* reached 50%, whereas there were no deaths in the control group (Supplementary Figure S2B). In addition, the 72-h culture supernatant of HN_xs01, either in the primary or secondary form, inhibited the growth of *H. armigera* larvae via using an oral route of infection (Table 1).

**TABLE 1:** Inhibition of *H. armigera* larval growth after 72 h-culture of different forms of strain HN_xs01.

	Sample number	AVG ±SD (cm)	SX*	CVIB** (cm)
CK	24	0.560 ± 0.054	0.011	0.395–0.650
Phase I	24	0.349 ± 0.061	0.013	0.220–0.440
Phase II	24	0.380 ± 0.062	0.013	0.275–0.485

*Std. Error mean.

*Cyclic variation of integrated backscatter.

CK, control. Phases I and II, indicate the primary and secondary forms of strain HN_xs01, respectively.

B16 melanoma cells were observed using phase contrast microscopy to visualize morphological changes in response to cultivation with HN_xs01. The results demonstrated that the cells of the control group (exposed to sterile LB) consisted of elongated, attached cells (Supplementary Figure S3B). In contrast, cells treated with the 72 h culture supernatants of the primary and secondary forms of HN_xs01 exhibited morphological alterations, including shrinkage and surface detachment, suggesting that the supernatants of strain HN_xs01 might induce cell apoptosis (Supplementary Figures S3C,S3D).

### Structure elucidation of cytotoxic rhabdopeptides

Three compounds (**1–3**) were purified from the crude extract of the strain HN_xs01. The molecular formula of **1** was determined to be C_31_H_54_N_5_O_4_ based on the protonated HRMS peak at *m*/*z* 560.41601 [M + H]^+^ (calculated for 560.4170) (Supplementary Figure S6). The chemical shifts of the NMR spectra indicated the presence of one phenethylamine, three N-methyl-valines, and one valine (Supplementary Table S1 and Supplementary Figures S5,S7–S10). The molecular formula of **2** was determined to be C_37_H_65_N_6_O_5_ based on the protonated HRMS peak at *m*/*z* 673.50119 [M + H]^+^ (calculated for 673.5011) (Supplementary Figure S6). The chemical shifts in the NMR spectra indicated the presence of one phenethylamine, four N-methyl-valines, and one valine (Supplementary Table S2, Supplementary Figures S5, S11–S14). In addition, the molecular formula of **3** was determined to be C_43_H_75_N_7_O_6_ based on the protonated HRMS peak at *m*/*z* 786.5868 [M + H]^+^ (calculated for 786.5852) (Supplementary Figure S6). The chemical shifts of the NMR spectra indicated the presence of one phenethylamine, five N-methyl-valines, and one valine (Supplementary Table S3, Supplementary Figure S5, S15–S18). The structures of **1, 2, 3** showed that they belonged to the rhabdopeptides, and the difference was the number of residues of the amino acid N-methyl-valine. The cytotoxic assays demonstrated that **3** had strong cytotoxic activity against A431 cells and K562 cells compared with **1** and **2**, with IC_50_ values of 2.5 μg/ml and 1.5 μg/ml, respectively (Table 2). Based on the anticancer activity results, we deduced that N-methyl-valine might enhance the cytotoxic bioactivity of compounds.

**TABLE 2:** *In vitro* cytotoxicity (IC_50_) of isolated compounds against A431 and K562 cells.

		IC_50_ [µg/mL]	
Compound	Formula	A431	K562
**1**	C_31_H_53_N_5_O_4_	>50	22.0
**2**	C_43_H_75_N_7_O_6_	ca. 40**	18.0
**3**	C_37_H_64_N_6_O_5_	2.5	1.5
**Zileuton**	C_11_H_12_N_2_O_2_S	7.7	7.1

“**” indicates branch cells.

A431 cells, human epithelial carcinoma cells; K562 cells, human chronic myelogenous leukemia cells.

### HN_xs01 targeted and retrained the growth of B16 tumors *in vivo*

Based on its wide growth characteristics and the potent antitumor activity of HN_xs01, we furtherly quested whether this strain could be as a novel antitumor-targeting strain *in vivo* or not. Two strategies of intravenous and intratumoral administrations of HN_xs01 to the C57BL/C mice bearing B16 tumor were performed. At the beginning, the maximum concentration of HN_xs01 administrated by intravenous injection to the healthy C57BL/C mice was investigated. The results demonstrated that high numbers of HN_xs01 (5 × 10^8^ CFU/100 μL) caused immediate death. Injection of 1 × 10^8^ CFU/100 μL resulted in 50% mortality in the mice. At a concentration of 5 × 10^7^ CFU/100 μL, all mice were still alive for 10 days (Supplementary Figure S4); thus, the results showed that four concentrations (1 × 10^6^ CFU/100 μL, 5 × 10^6^ CFU/100 μL, 1 × 10^7^ CFU/100 μL, and 5 × 10^7^ CFU/100 μL) were safe, and we used an intravenous injection of 5 × 10^7^ CFU/100 μL to investigate the inhibition of HN_xs01 in the C57BL/C B16 tumor-bearing model.

**FIGURE 2:**
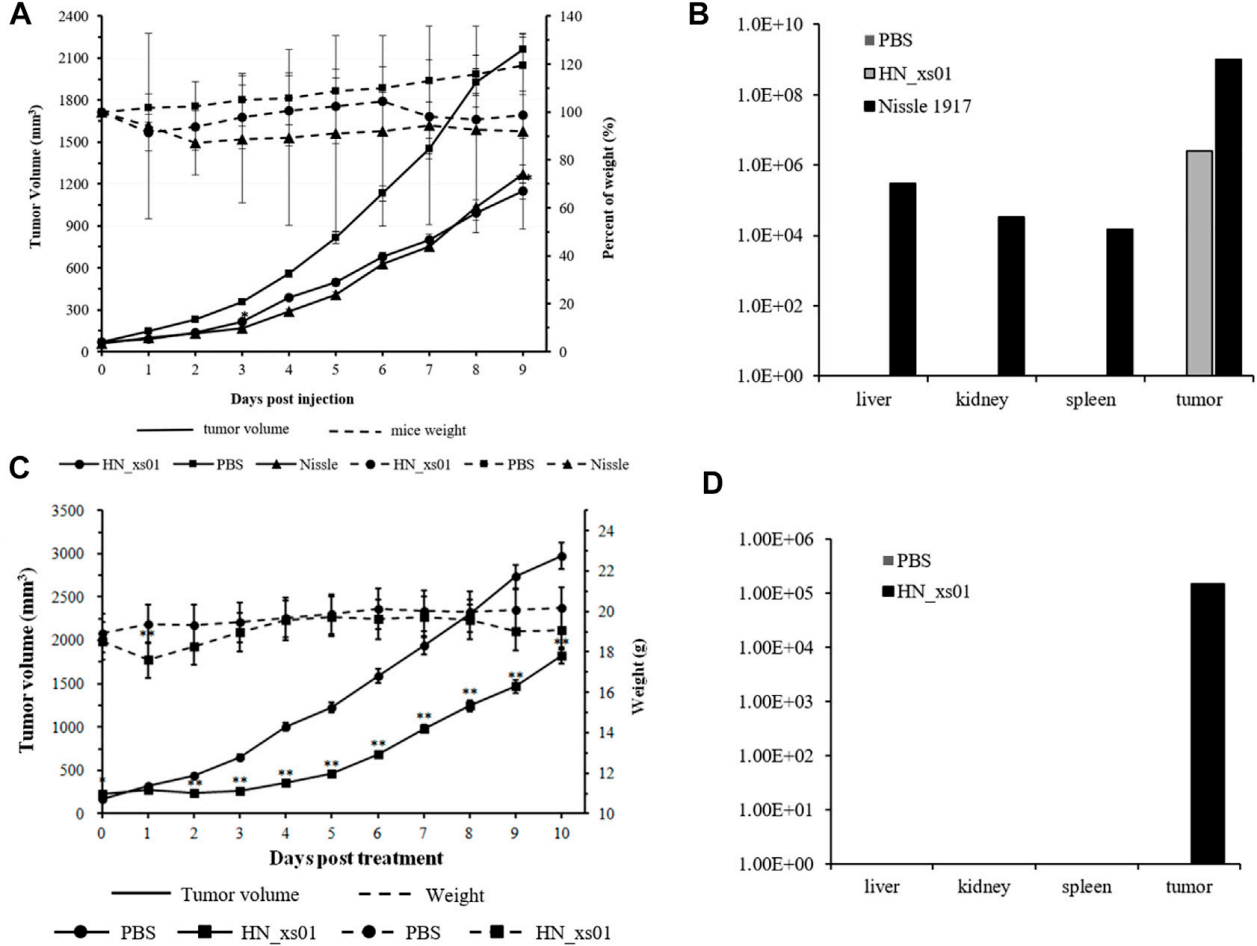
The effect and distribution of HN_xs01 on tumor growth and organ colonization following intravenous and intratumoral injections. **(A)** Mice bearing B16 tumors were injected intravenously with PBS, 2 × 10^6^ CFU/100 μl of *E. coli* Nissle 1917, or 5 × 10^7^ CFU/100 μl of HN_xs01, respectively. Tumor volume (mm^3^) and percent of mice weight (%) were measured mice. The antitumor activity of HN_xs01 was similar to that of *E. coli* Nissle 1917 by intravenous administration. Tumors were significantly inhibited by intravenous injection of HN_xs01. **(B)** Bacterial distribution in organs was calculated by CFU/g by intravenous administration. Strain HN_xs01 was undetectable in the liver, kidney, and spleen, but small amounts of *E. coli* Nissle 1917 were detected in other organs compared to the tumor area. **(C)** Mice bearing B16 tumors were treated by intratumoral injection of PBS or 1 × 10^8^ CFU/100 μl of HN_xs01. Tumors were significantly inhibited by intratumoral injection of HN_xs01. **(D)** Bacterial distribution in organs was calculated by CFU/g *via* intratumoral administration. Strain HN_xs01 strictly colonized the tumor region. Data are shown for n = 4 mice per group by intravenous injection and n = 7 mice per group for intratumoral injection.

To illustrate the tumor-specific colonization and repression of HN_xs01, we compared HN_xs01 with the probiotic *E. coli* Nissle 1917, which had efficient tumor colonization (Stritzker et al., 2007). The number of the intravenous injections of the strain HN_xs01 and the amount of *E. coli* Nissle 1917 was 5 × 10^7^ CFU/100 μL and 2 × 10^6^ CFU/100 μL, respectively, according to the above result and previous study. The results showed that both strains notably delayed tumor growth compared with the control group, suggesting no obvious disparity in antitumor activity between HN_xs01 and Nissle 1917 (Figure 2A). However, antitumor activity was more sustainable in the preliminary stage after the administration of HN_xs01. Eight days after the administration of bacteria, the HN_xs01 group exhibited preferential inhibition of tumor growth compared with the Nissle 1917 group. Additionally, we observed obvious differences in body weight after the injection of PBS, HN_xs01, or Nissle 1917. The control group demonstrated slow growth, whereas the body weight of mice treated with Nissle 1917 decreased continuously. Interestingly, the body weight of mice decreased slightly after the first injection of HN_xs01, and 3 days later, the weight began to recover. We performed a second injection 6 days after the first, and a similar phenomenon was observed (Figure 2A). Thus, strain HN_xs01 had no obvious effect on the mice growth, suggesting that it is less toxic than Nissle 1917. Smaller amounts of *E. coli* Nissle 1917 were detected in the liver (3 × 10^5^ CFU/g), kidney (3.4 × 10^4^ CFU/g), and spleen (1.5 × 10^4^ CFU/g) than in the tumor (1 × 10^9^ CFU/g). However, strain HN_xs01 was concentrated in the tumor area and undetectable in other organs (Figure 2B). Surprisingly, the number of Nissle 1917 bacteria in the tumor area was higher than that of HN_xs01, we deduced that HN_xs01 might be easily eliminated by the immune system of mice compared to Nissle 1917. To sum up, HN_xs01 had stronger tumor-specific colonization than Nissle 1917 in the aspect of the intravenous administration, indicating that HN_xs01 was a promising strain for intravenous tumor treatment.

**FIGURE 3:**
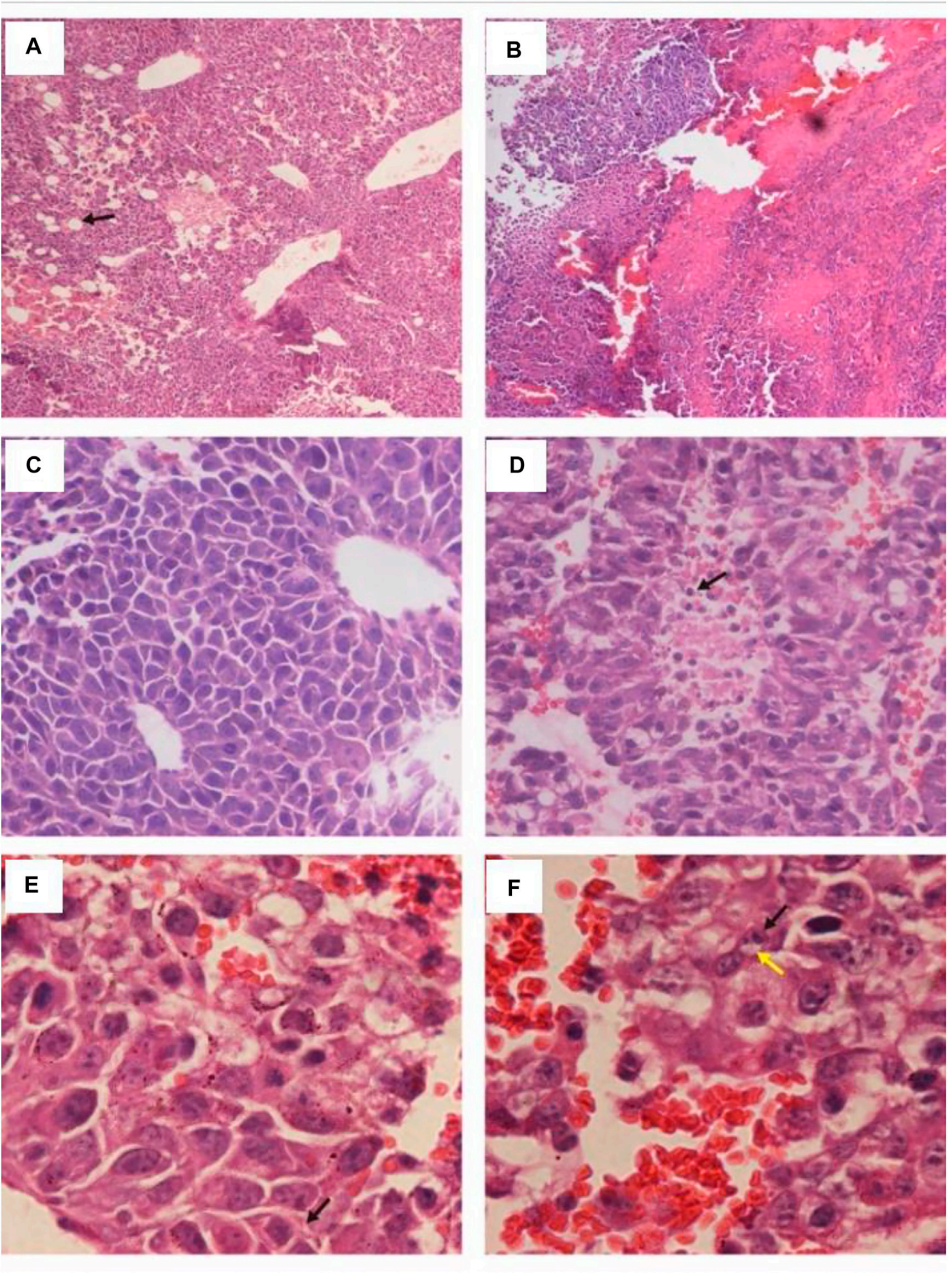
Histology of B16 tumors injected with HN_xs01 or PBS. C57BL/C mice bearing B16 tumors were injected intratumorally with 1 × 10^8^ CFU/100 μl of HN_xs01, and the tumors were examined by H&E staining at 6 days post-treatment. The left panels show results of PBS treatment, and the right panels show results of HN_xs01 treatment. The areas of B16 melanoma is shown under 100 × low magnification in **(A,B)**. Enlargement of areas of B16 melanoma is shown in panels **(C–F)**. The arrows in **(A,D)** indicate areas of blood vessels and dividing cells. The black and yellow arrows in **(E,F)** indicate shrunken chromatin and physalides, respectively.

In the C57BL/C B16 tumor-bearing model, treatment with HN_xs01 significantly delayed tumor growth through intratumoral administration as well. Ten days after administration, the tumor volume in the control group was 1.63 fold greater than that in the treatment group. Nevertheless, the weight of mice treated with HN_xs01 initially decreased and recovered after 3 days, which suggested that strain HN_xs01 had a transient negative effect on the mice (Figure 2C). At sacrifice, no bacteria were present in the liver, kidney, or spleen, and strain HN_xs01 strictly colonized the tumor area (Figure 2D). These data revealed that HN_xs01 exhibited significant antitumor activity and tumor targeting following intratumoral injection. In addition, the B16 tumor of the treatment group appeared the conspicuous necrotic areas, fewer blood vessels, sparse cells, chromatin-forming physalides, and undivided nuclei via H&E staining comparing with no abnormalities and maintained their division of the tumor cells in the control group (Figures 3A–F). The differences of the tumor morphology between the PBS and HN_xs01 treatment groups indicate that HN_xs01 inhibits tumor growth by inhibiting blood vessel proliferation and inducing cell apoptosis. Thus, HN_xs01 inhibits tumor growth through multiple mechanisms.

### HN_xs01 significantly restrained the pulmonary metastasis of B16 tumors

**FIGURE 4:**
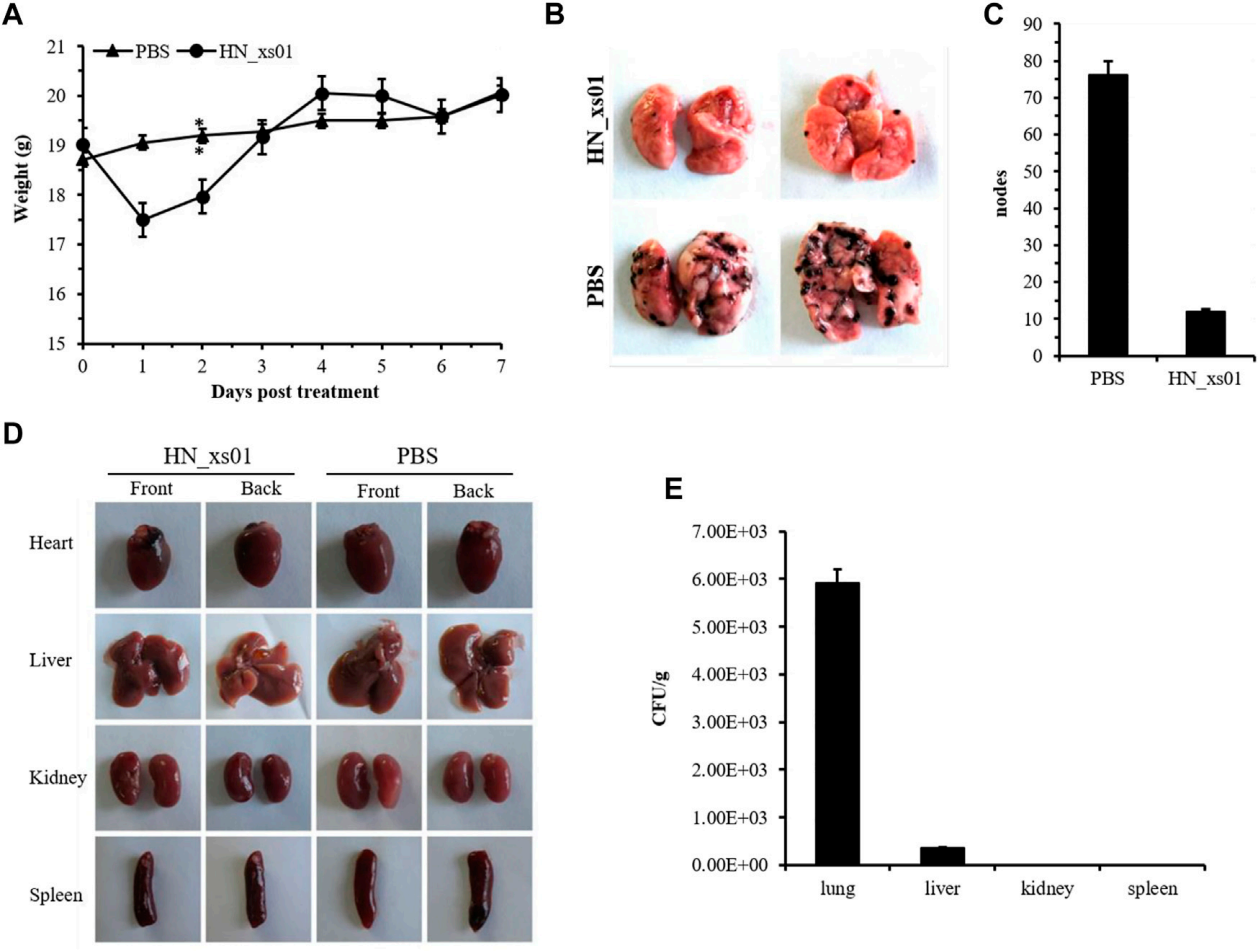
The effect of HN_xs01 on pulmonary metastasis of B16 tumors. **(A)** Mice with B16 tumors were injected intravenously with PBS or 5 × 10^7^ CFU/100 μl of HN_xs01, and body weight was measured daily. **(B)** The nodes distribution in the lungs of the PBS and HN_xs01 groups. After 1 week, the lungs, hearts, livers, kidneys, spleens, and tumors were isolated. **(C)** The nodes number in the lungs of the PBS and HN_xs01 groups. **(D)** The tumor nodes distribution in the heart, liver, kidney, and spleen tissues between the PBS and HN_xs01 groups. **(E)** Bacterial distribution in organs was calculated by CFU/g. Strain HN_xs01 mostly colonized the lung region.

Body weight was slightly affected by the intravenous injection of HN_xs01, but the weight returned to normal 3 days post-injection (Figure 4A). After 7 days, the organisms of the control and treatment groups were taken out. We found that the tumor nodes were detected in the lungs of the PBS and HN_xs01 treatment groups, and the number of nodes on the lung surfaces of mice from the PBS group was 6.35-fold greater than that in the HN_xs01 group, but not undetected in the heart, liver, kidney and spleen tissues, confirming that the B16 tumor model spontaneously metastasizes from tumors to the lungs (Figures 4B–D). At the treatment group, the number of the bacteria HN_xs01 in the lung (6 × 10^3^ CFU/g) of mice was significantly greater than that in the liver (353 CFU/g), whereas no bacteria were detected in the kidney or spleen tissues (Figure 4E). These data demonstrated that HN_xs01 could efficiently inhibit pulmonary metastasis of mouse melanoma.

### Intratumoral administration of HN_xs01 efficiently prolonged mouse survival time

**FIGURE 5:**
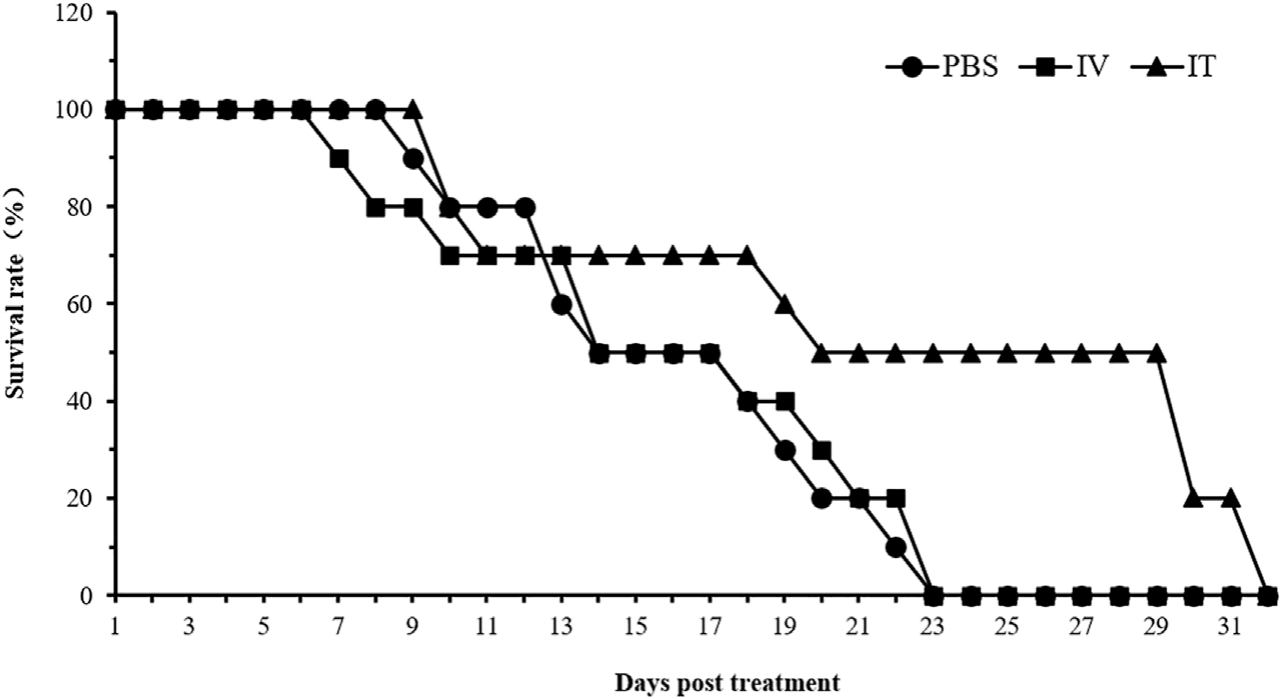
The effect of HN_xs01 on mouse survival. C57BL/6 mice were administered with strain HN_xs01 intravenously (5 × 10^7^ CFU/100 μl) and intratumorally (1 × 10^8^ CFU/100 μl) for 32 days, respectively. The control group received an equivalent volume of PBS. There were 10 mice per group, and the mice were monitored for 32 days. IV: intravenous injection, IT: intratumoral injection.

C57BL/C mice bearing B16 tumors were administered with 5 × 10^7^ CFU/100 μL of strain HN_xs01 intravenously, 1 × 10^8^ CFU/100 μL of strain HN_xs01 by intratumorally, and PBS (control). The intratumoral injection significantly extended the mouse survival time; the mortality rate reached 50% 20 days after injection and 100% by day 32 post-injection (Figure 5). In contrast, the mortality rates of the PBS group and intravenous injection groups reached 100% after 23 days. Thus, the intratumoral injection of HN_xs01 was significantly more effective than the intravenous injection in prolonging mouse survival time.

## Discussion

In our study, the bacterial strain HN_xs01 was identified as *X. stockiae* and found to have multiple high-efficiency biological activities. *Xenorhabdus* strains can spontaneously produce two physiological forms (primary and secondary) *in vitro*. The primary form can produce several antibiotics and various proteins; however, these properties are greatly reduced or absent in the secondary form (Gualtieri et al., 2009). Nonetheless, a secondary form, such as the hypopus, is produced during *in vitro* monoxenic culture of nematodes, where the bacterium is detrimental to the final yield of nematodes (Akhurst, 1980). There were significant differences in the growth curves and the optimal pH for growth between the primary and secondary forms. HN_xs01 grew over a wide temperature range, indicating that this strain was heat-resistant. It also grew well at pH 6.5 and pH 8.0 owing to its original living environment (i.e., the intestinal vesicles in nematodes and hemocoel of insects) (Bird and Akhurst, 1983).

*Xenorhabdus* strains are rich resources of active natural compounds. However, research on symbiotic bacteria of entomopathogenic nematodes is relatively new. Here, we tested the antimicrobial, insecticidal, and antitumor activities of the strain HN_xs01, which has broad-spectrum antimicrobial properties. *S. aureus* and *S. typhimurium* were highly sensitive to HN_xs01 in antimicrobial bioassays. In previous studies, comparative analysis of the antimicrobial activity of *Xenorhabdus* species on other bacteria suggested that antibiotics produced by *Xenorhabdus* were effective against animal and plant pathogens (Furgani et al., 2008; Fodor et al., 2010). Furthermore, *Xenorhabdus* strains have potential antifungal activity relevant to agriculture (Fang et al., 2011; Tobias et al., 2018). Strain HN_xs01 exhibited potent activity against *H. armigera* larvae when injected. However, its oral insecticidal activity was poor and only inhibited insect growth. Therefore, our findings suggest that the toxins of HN_xs01 are presumably essential for bacteria to rapidly kill infected insects, and act preferentially when injected, similarly to *Xenorhabdus* (Sergeant et al., 2006). Additionally, *Xenorhabdus* possesses significant pathogenic activity against some important insect pests of commercial crops, such as lepidopteran, dipteran, and coleopteran, and is considered a neotype biological insecticide after *B. thuringiensis* (Burnell and Stock, 2000; Chattopadhyay et al., 2004; Georgis et al., 2006). A high-efficiency “Bt-Plus” insecticide has been reported to enhance the insecticidal activity of *B. thuringiensis* by mixing it with broth cultures of the *X. nematophila* primary form, which suppresses target insect immunity (Eom et al., 2014).

*In vitro* cytotoxicity assays using extracts of strain HN_xs01 exhibited antitumor activities, and purified compounds **1**, **2**, and **3**, belonging to the rhabdopeptide/xenortide peptide class (RXPs) were the same with the rhabdopeptides elucidated from an extract mixture based on labeling and MS experiments previously (Reimer et al., 2013; Cai et al., 2017; Shi and Bode, 2018). However, the structures of compounds **1**, **2**, **3** were first elucidated by NMR. In addition, three of the seven rhabdopeptides discovered by the Yu group were known rhabdopeptides, and the other four novel rhabdopeptides included two valines (Bi et al., 2018). Moreover, the potent antitumor activities of **3** against human epithelial carcinoma cell line A431 and human chronic myelogenous leukemia cell line K562 reveal the rhabdopeptides could be promising drugs for the tumor chemotherapy. Notably, the efficient tumor-specific colonization and replication of HN_xs01 in the tumor regions compared with the probiotics *E. coli* Nissle 1917, combined with HN_xs01 producing the antitumor compounds contribute strain HN_xs01 to restrain B16 solid tumors. Bacteria can induce cell apoptosis through multiple mechanisms, including the stimulation of immune cells, competition for extracellular nutrients, and induction of apoptotic signal transduction pathways following intracellular accumulation (Ganai et al., 2011). Our analyses indicated that active secondary metabolites produced by strain HN_xs01 also contribute to tumor cell apoptosis. Moreover, HN_xs01 significantly restrained the pulmonary metastasis of B16 tumors. These characteristics predict that HN_xs01 could be a novel bacterial tumor-targeting strain.

The application of bacteria in cancer therapy has been presented as a novel approach, although the potential of this approach has been described before the development of conventional therapies (Baban et al., 2010; Liu et al., 2014). Tumor regression has been observed in cancer patients suffering from infections with *Clostridium* or *Streptococcus pyogenes* (Felgner et al., 2016). Subsequently, many strains of various genera have been tested for their potential in cancer therapy using various animal models. Spores of *Clostridium* only germinate in the absence of oxygen and cannot reach anaerobic regions; therefore, metastases or small tumors without necrotic areas might not be targeted by this approach. Facultative anaerobic bacteria, such as *S. typhimurium,* can grow under aerobic conditions but potentially disseminates to healthy tissues (Choe et al., 2014; Hoffman and Zhao, 2014). Although bacteria can be modified for use in clinical studies, their use presents many disadvantages that cannot be avoided in cancer therapy. To solve this problem, suitable strains are acquired by passaging *in vitro* or *in vivo* or by deleting targeted genes using molecular techniques. Our analysis showed that the intratumoral administration of HN_xs01 was more beneficial to prolong the mouse survival than intravenous administration. We deduced that HN_xs01 might spread to healthy tissue liver of mice via intravenous injection resulting in liver damage. Thus, HN_xs01 need be engineered furtherly to make it be suitable for clinical cancer therapy. Gene-based anti-angiogenic therapy has been used in conjunction with other approaches to decrease angiogenesis, such as bifidobacterial expression of endostatin genes (Baban et al., 2010; Cummins and Tangney, 2013). Engineering the strain HN_xs01 to successfully express cytotoxic agents such as glidobactin and luminmide (data not shown), is an efficient strategy for enhancing its antitumor properties, which suggests that HN_xs01 could be a potential chassis for the other active antitumor resources.

In conclusion, the primary and secondary forms of the identified bacterium *X. stockiae* HN_xs01 exhibit potent and diverse biological activities. The efficient tumor-specific colonization and inhibition of *X. stockiae* HN_xs01 to B16 solid tumors and pulmonary metastasis *in vivo* manifest it be a novel bacterial tumor-targeting strain for cancer therapy in the future.

## Data Availability

The 16S rRNA gene sequence of strain HN_xs01 was submitted to the GenBank database, accession number HQ840745.
