# Supplementary material for: A novel tumor-targeting strain of Xenorhabdus stockiae exhibits potent biological activities
Source: Front Bioeng Biotechnol. 2022 Sep 7;10:984197. doi: 10.3389/fbioe.2022.984197 (PMC9490112; doi:10.3389/fbioe.2022.984197)
Supplement: Supplementary file 1 [file Table1.DOCX]

Supplementary Material

**TABLE S1** ^1^H (700 MHz) and ^13^C NMR (700 MHz) analysis of **1** in MeOD-*d*_4_.

**TABLE S2** ^1^H (700 MHz) and ^13^C NMR (700 MHz) analysis of **2** in MeOD-*d*_4_.

**TABLE S3** ^1^H (700 MHz) and ^13^C NMR (700 MHz) analysis of **3** in MeOD-*d*_4_.

**FIGURE S1.** Growth characteristics of strain HN_xs01 in LB broth.

**FIGURE S2.** Antimicrobial and insecticidal activities of the different forms of strain HN_xs01.

**FIGURE S3.** Cytotoxic activity of 72-h culture of strain HN_xs01 *in vitro*.

**FIGURE S4.** Tolerance of C57BL/C mice for HN_xs01 administered by intravenous injection

**FIGURE S5.** Structures of compounds **1**, **2**, and **3**.

**FIGURE S6.** High-resolution ESI-MS and MSMS of three linear rhabdopeptides.

**FIGURE S7.** ^1^H-NMR spectra of compound **1** in MeOD-d_4_ at 700 MHz.

**FIGURE S8.** HSQC-NMR spectra of compound **1** in MeOD-*d_4_* at 700 MHz.

**FIGURE S9.** HMBC-NMR spectra of compound **1** in MeOD-*d_4_* at 700 MHz.

**FIGURE S10.** COSY-NMR spectra of compound **1** in MeOD-*d_4_* at 700 MHz.

**FIGURE S11.** ^1^H-NMR spectra of compound **2** in MeOD-d_4_ at 700 MHz.

**FIGURE S12.** HSQC-NMR spectra of compound **2** in MeOD-*d_4_* at 700 MHz.

**FIGURE S13.** HMBC-NMR spectra of compound **2** in MeOD-*d_4_* at 700 MHz.

**FIGURE S14.** COSY-NMR spectra of compound **2** in MeOD-*d_4_* at 700 MHz.

**FIGURE S15.** ^1^H-NMR spectra of compound **3** in MeOD-d_4_ at 700 MHz.

**FIGURE S16.** HSQC-NMR spectra of compound **3** in MeOD-*d_4_* at 700 MHz.

**FIGURE S17.** HMBC-NMR spectra of compound **3** in MeOD-*d_4_* at 700 MHz.

**FIGURE S18.** COSY-NMR spectra of compound **3** in MeOD-*d_4_* at 700 MHz.

**Material and methods**

**Bacterial growth characteristics**

*Xenorhabdus stockiae* strain HN_xs01 was streaked on NBTA plates and incubated at 30^o^C for 36 h. Blue and red colonies were inoculated separately into 10 ml LB broth and incubated for 36 h at 30^o^C on a rotary shaker at 200 rpm. Afterwards, 1 mL culture was transferred into 100 ml fresh LB broth and shaken for 108 h at 30^o^C at 200 rpm. Then OD_600_ and culture pH of the two phases of HN_xs01 were measured. The optimal pH and temperature for culturing of HN_xs01 were also measured by OD_600_ over a 12-h period.

**Biological activity assays**

Overnight cultures were transferred into 100 mL fresh LB broth at a ratio of 1:100 and incubated at 30^o^C on a rotary shaker at 200 rpm. Samples were taken out after incubation for 6 h, 9 h, 12 h, 24 h, 48 h, and 72 h and centrifuged (10 min, 10^o^C at 11000 rpm) to separate the bacterial cells from the supernatants. Then, the supernatants were filtered through 0.22 µm filter nozzles, and 10-times (10 ×) concentrated solutions from the filtrates were acquired using a Speed Vac Concentrator System (Thermo Fisher). The antimicrobial activities of 20 µL concentrated filtrates from the 6 h, 9 h, 12 h, 24 h, and 48 h cultures were examined by agar diffusion assays using paper discs (6 mm diameter) against a set of test organisms, including *Enterobacter aerogenes*, *Bacillus subtilis*, *Lysinibacillus sp.*, *Staphylococcus aureus*, *Salmonella typhimurium*, and *Escherichia coli*.

Insecticidal activity of 10 µL concentrated filtrates from the 24 h, 48 h, and 72 h cultures were tested against *Helicoverpa armigera* (10 insects for each group) by injection. The bollworms were incubated at 30^o^C and observed for mortality at set intervals. In addition, the oral insecticidal activity was tested using 5 mL of 72 h culture (or an equivalent volume of LB broth for the control group) mixed with 25 mL artificial broth; the mixture was dispensed onto 72 bioassay disks and dried for 15 min. Next, 72 *H. armigera* larvae were individually placed onto the disks. After incubating at 28^o^C for 72 h in a constant temperature incubator, lengths of the larvae were measured, and the data were analyzed with SPSS 17.0 software.

The cytotoxic activity of HN_xs01 against B16 melanoma cells was evaluated *in vitro*. Briefly, 100 µL of cell suspension was cultured in 96-well plates, at a final concentration of 1 × 10^6^ cells per well, at 37^o^C with 5% CO_2_ for 9 h. 72 h culture of the primary form and 72 h culture of the secondary form was added to the wells, and the equivalent volume of PBS was added to the wells. After cells treated for 24 h, the morphology of B16 cells was observed.

**Chemical analysis: General information**

LCHR-MS and MS/MS analysis. All measurements were performed on a Dionex Ultimate 3000 RSLC (Thermo, Dreieich, Germany) comprising a high pressure gradient pump (HPG-3400RS) with a 150 μL mixing chamber. A BEH C18, 100 × 2.1 mm, 1.7 μm dp column (Waters, Eschborn, Germany) was used to separate 2 μl sample by a linear gradient from (A) H_2_O + 0.1% HCOOH to (B) CH_3_CN + 0.1% HCOOH at a flow rate of 600 μL min^-1^ at 45°C. The gradient is initiated by a 0.5 min isocratic step at 5% B, followed by an increase to 95% B in 18 min to end up with a 2 min step at 95% B before reequilibration under the initial conditions (LC-system I) or by a linear gradient with the same initiation, plateau and reequilibration steps like for system I with a gradient length of 30 min (LC-system II) or a gradient length of 9 min (LC-system III). The LC flow is split to 75 μL min^-1^ before entering the maXis 4G hr-QqToF mass spectrometer (Bruker Daltonics, Bremen, Germany) using the Apollo ESI source. The ion source parameters are: capillary, 4000 V; endplate offset, -500 V; nebulizer, 1 bar; dry gas, 5 l/min; and dry gas temperature, 200°C. Ion transfer parameters are: funnel RF, 350 Vpp; multipole RF, 400 Vpp; quadrupole ion energy, and 5 eV at low m/z 200. Collision cell is set to 8 eV with a collision RF of 2500 Vpp in full scan mode. Ion cooler settings are: transfer time, 90 μs; ion cooler RF, 120 Vpp; and pre puls storage, 5 μs. Mass spectra are acquired in centroid mode ranging from 150-2500 m/z at a 2 Hz scan rate in full scan positive ESI mode. Each measurement is started with the injection of a 20 μL plug of basic sodium formate solution introduced by a loop switched into the flow path. The resulting peak is used for automatic internal m/z calibration. In addition, a lock mass (Agilent Chip Cube High Mass HP-1221, Art.# G1982-85001) is used for recalibration of single spectra.

MS/MS settings. Minimum precursor intensity is set to 10,000. Full scan spectra are acquired at 2 Hz followed by MSMS spectra acquisition at variable scan speed ranging from 1.5 to 5 Hz being a function of precursor intensity. CID energy varies linearly from 30, 35 to 45 eV with respect to the precursor masses of 300, 600, 1000 *m*/*z*. The ion cooler is set to ramp collision energy (90-120% of the set value) and ion cooler RF from 120 to 80 Vpp for every MSMS scan. The precursor list is evaluated every 2 seconds to assign the upcoming precursors and precursors were moved to an exclusion list for 0.2 min after two spectra were measured (typical chromatographic peak width was 0.10-0.15 min).

NMR spectroscopical analysis. NMR spectra were recorded on a 500 MHz Avance III (UltraShield) spectrometer or on a 700 MHz Avance III (Ascend) spectrometer from Bruker BioSpin GmbH, equipped each with a 5 mm TXI cryoprobe at 298 K. Chemical shift values of ^1^H- and ^13^C-NMR spectra are reported in ppm relative to the residual solvent signal given as an internal standard. ^13^C-signals were assigned via 2D-CH and CCH or CNH correlations (HSQC and HMBC). Multiplicities are described using the following abbreviations: s = singlet, d = doublet, t = triplet, q = quartet, m = multiplet, b = broad; coupling constants are reported in Hz.

**TABLE S1** ^1^H (700 MHz) and ^13^C NMR (700 MHz) analysis of **1** in MeOD-*d*_4_.

| pos. | d_H_, mult (*J* in Hz) | d_C_^*^ | COSY correlations | HMBC correlations |
| --- | --- | --- | --- | --- |
| **Phenethylamine** |  |  |  |  |
| 1 | 3.45 m, 3.41 m | 41,41 | 2 | 3, 1.2 |
| 2 | 2 x 2.77 | 36,32 | 1 | 4 |
| 3 |  | 140,2 |  |  |
| 2 x 4 | 7.20 d (7.55) | 129,58 | 5 | 2 |
| 2 x 5 | 7.27 dd (7.55, 7.55) | 129,32 | 4, 6 | 3 |
| 6 | 7.18 m | 127,22 | 5 |  |
| N-H | 7.99 m |  | 1 | 1,2 |
|  |  |  |  |  |
| **N-methyl-valine 1** |  |  |  |  |
| 1,2 |  | 171,48 |  |  |
| 2,2 | 4.59 d (7.91) | 63,45 | 3,2 | 1.2, N-methyl, 1.3 |
| 3,2 | 2.19 m | 27,28 | 2.2, 4.2, 5.2 | 1,2 |
| 4,2 | 3 x 0.87 d (6.54) | 19,44 | 3,2 |  |
| 5,2 | 3 x 0.75 d (6.69) | 18,69 | 3,2 |  |
| N-methyl | 3 x 3.06 s | 31,28 |  | 2.2, 1.3 |
|  |  |  |  |  |
| **N-methyl-valine 2** |  |  |  |  |
| 1,3 |  | 172,52 |  |  |
| 2,3 | 5.19 d (11.08) | 59,28 | 3,3 | 1.3, N-methyl, 1.4 |
| 3,3 | 2.32 m | 28,6 | 2.3, 4.3, 5.3 | 1,3 |
| 4,3 | 3 x 0.86 d (6.43) | 19,44 | 3,3 |  |
| 5,3 | 3 x 0.81 d (6.78) | 18,82 | 3,3 |  |
| N-methyl | 3 x 3.16 s | 30,97 |  | 2.3, 1.4 |
|  |  |  |  |  |
| **Valine 3** |  |  |  |  |
| 1,4 |  | 173,72 |  |  |
| 2,4 | 4.75 d (5.32) | 56,35 | 3,4 | 1.4, 1.5 |
| 3,4 | 2.07 m | 31,39 | 2.4, 4.4, 5.4 | 1,4 |
| 4,4 | 3 x 0.96 d (6.90) | 19,42 | 3,4 |  |
| 5,4 | 3 x 1.02 d (18.25) | 18,25 | 3,4 |  |
|  |  |  |  |  |
| **N-methyl-valine 4** |  |  |  |  |
| 1,5 |  | 167,51 |  |  |
| 2,5 | 3.68 d (18.81) | 67,88 | 3,5 | 1.5, N-methyl |
| 3,5 | 2.17 m | 31,28 | 2,5 | 1,5 |
| 4,5 | 3 x 1.05 d (6.99) | 18,32 | 3,5 |  |
| 5,5 | 3 x 1.01 d (6.90) | 18,23 | 3,5 |  |
| N-methyl | 3 x 2.65 s | 32,91 |  | 1,5 |

pos. indicates position, multi indicates multiple.

**TABLE S2** ^1^H (700 MHz) and ^13^C NMR (700 MHz) analysis of **2** in MeOD-*d*_4_.

| pos. | d_H_, mult (*J* in Hz) | d_C_^*^ | COSY correlations | HMBC correlations |
| --- | --- | --- | --- | --- |
| **Phenethylamine** |  |  |  |  |
| 1 | 3.46 m, 3.41 m | 41,38 | 2, N-H | 3, 1.2 |
| 2 | 2 x 2.77 m | 36,26 | 1 | 3, 4 |
| 3 |  | 140,09 |  |  |
| 2 x 4 | 7.20 d (7.76) | 129,55 | 5 | 6 |
| 2 x 5 | 7.27 dd (7.61,7.61) | 129,34 | 4, 6 | 3 |
| 6 | 7.18 m | 127,22 | 5 | 4 |
| N-H | 7.97 m |  | 2 | 1,2 |
|  |  |  |  |  |
| **N-methyl-valine 1** |  |  |  |  |
| 1,2 |  | 171,66 |  |  |
| 2,2 | 4.57 d (11.09) | 63,47 | 3,2 | 1.2, N-methyl, 1.3 |
| 3,2 | 2.21 m | 27,32 | 2.2, 4.2, 5.2 | 1,2 |
| 4,2 | 3 x 0.86 d (640) | 19,57 | 3,2 |  |
| 5,2 | 3 x 0.74 d (6.69) | 18,58 | 3,2 |  |
| N-methyl | 3 x 3.04 s | 31,15 |  | 2.2, 1.3 |
|  |  |  |  |  |
| **N-methyl-valine 2** |  |  |  |  |
| 1,3 |  | 172,25 |  |  |
| 2,3 | 5.17 d (10.72) | 59,38 | 3,3 | 1.3, N-methyl, 1.4 |
| 3,3 | 2.30 m | 28,4 | 2.3, 4.3, 5.3 | 1,3 |
| 4,3 | 3 x 0.88 d (6.50) | 19,57 | 3,3 |  |
| 5,3 | 3 x 0.78 d (6.77) | 18,58 | 3,3 |  |
| N-methyl | 3 x x3.05 s | 31,15 |  | 2.3, 1.4 |
|  |  |  |  |  |
| **N-methyl-valine 3** |  |  |  |  |
| 1,4 |  | 172,01 |  |  |
| 2,4 | 5.20 d (10.72) | 59,41 | 3,4 | 1.4, N-methyl, 1.6 |
| 3,4 | 2.33 m | 28,4 | 2.4, 4.4, 5.4 | 1,4 |
| 4,4 | 3 x 0.87 d (6.45) | 19,57 | 3,4 |  |
| 5,4 | 3 x 0.79 d (6.74) | 18,58 | 3,4 |  |
| N-methyl | 3 x 3.16 s | 30,94 |  | 2.4, 1.5 |
|  |  |  |  |  |
|  |  |  |  |  |
| **Valine 4** |  |  |  |  |
| 1,5 |  | 173,76 |  |  |
| 2,5 | 4.77 d (7.65) | 56,34 | 3,5 | 1.5, 1.6 |
| 3,5 | 2.08 m | 31,13 | 2.5, 4.5, 5.5 |  |
| 4,5 | 3 x 1.02 d (6.71) | 18,24 | 3,5 |  |
| 5,5 | 3 x 0.99 d (6.77) | 19,59 | 3,5 |  |
|  |  |  |  |  |
| **N-methyl-valine 5** |  |  |  |  |
| 1,6 |  | 167,49 |  |  |
| 2,6 | 3.68 d (5.31) | 67,92 | 3,6 | 1.6, N-methyl |
| 3,6 | 2.17 m | 31,13 | 2.6, 4.6, 5.6 | 1,6 |
| 4,6 | 3 x 1.05 d (6.94) | 18,24 | 3,6 |  |
| 5,6 | 3 x 1.01 d (6.88) | 18,24 | 3,6 |  |
| N-methyl | 3 x 2.65 s | 32,93 |  | 2,6 |

pos. indicates position, multi indicates multiple.

**TABLE S3** ^1^H (700 MHz) and ^13^C NMR (700 MHz) analysis of **3** in MeOD-*d*_4_.

| pos. | d_H_, mult (*J* in Hz) | d_C_^*^ | COSY correlations | HMBC correlations |
| --- | --- | --- | --- | --- |
|  |  |  |  |  |
| **Phenethylamine** |  |  |  |  |
| 1 | 3.47 m, 3.41 m | 41,43 | 2, N-H | 3, 1.2 |
| 2 | 2 x 2.77 m | 36,36 | 1 | 4 |
| 3 |  | 140,16 |  |  |
| 2 x 4 | 7.20 d (7.78) | 129,62 | 5 | 2 |
| 2 x 5 | 7.26 dd (7.42, 7.42) | 129,4 | 4, 6 | 3 |
| 6 | 7.18 m | 127,28 | 5 |  |
| N-H |  |  | 1 | 1,2 |
|  |  |  |  |  |
| **N-methyl-valine 1** |  |  |  |  |
| 1,2 |  | 171,57 |  |  |
| 2,2 | 4.57 d (11.11) | 63,59 | 3,2 | 1.2, N-methyl, 1.3 |
| 3,2 | 2.21 m | 27,42 | 2.2, 4.2, 5.2 | 1,2 |
| 4,2 | 3 x 0.87 m | 19,64 | 3,2 |  |
| 5,2 | 3 x 0.74 d (6.72) | 18,51 | 3,2 |  |
| N-methyl | 3 x 3.05 s | 31,13 |  | 2.2, 1.3 |
|  |  |  |  |  |
| **N-methyl-valine 2** |  |  |  |  |
| 1,3 |  | 172,34 |  |  |
| 2,3 | 5.16 d (10.78) | 59,46 | 3,3 | 1.3, N-methyl, 1.4 |
| 3,3 | 2.32 m | 28,39 | 1.3, 4.3, 5.3 | 2.3, 1.3 |
| 4,3 | 3 x 0.87 m | 19,64 | 3,3 |  |
| 5,3 | 3 x 0.78 d (6.78) | 18,51 | 3,3 |  |
| N-methyl | 3 x 3.04 s | 31,13 |  | 2.3, 1.4 |
|  |  |  |  |  |
| **N-methyl-valine 3** |  |  |  |  |
| 1,4 |  | 171,98 |  |  |
| 2,4 | 5.20 d (5.19) | 59,46 | 3,4 | 1.4, N-methyl, 1.5 |
| 3,4 | 2.32 m | 28,39 | 2.4, 4.4, 5.4 | 1,4 |
| 4,4 | 3 x 0.87 m | 19,64 | 3,4 |  |
| 5,4 | 3 x 0.80 d (6.81) | 18,51 | 3,4 |  |
| N-methyl | 3 x 3.03 | 31,13 |  | 2.4, 1.5 |
|  |  |  |  |  |
| **N-methyl-valine 4** |  |  |  |  |
| 1,5 |  | 171,98 |  |  |
| 2,5 | 5.21 d (5.19) | 59,46 | 3,5 | 1.5, N-methyl, 1.6 |
| 3,5 | 2.32 m | 28,39 | 2.5, 4.5, 5.5 | 1,5 |
| 4,5 | 3 x 0.87 m | 19,64 | 3,5 |  |
| 5,5 | 3 x 0.77 d (6.66) | 18,51 | 3,5 |  |
| N-methyl | 3 x 3.15 s | 30,97 |  | 2.5, 1.6 |
|  |  |  |  |  |
| **Valine 5** |  |  |  |  |
| 1,6 |  | 173,83 |  |  |
| 2,6 | 4.77 d (7.55) | 56,39 | 3,6 | 1.6, N-methyl, 1.7 |
| 3,6 | 2.09 m | 31,35 | 2.6, 4.6, 5.6 | 1,6 |
| 4,6 | 3 x 0.99 d (6.78) | 19,68 | 3,6 |  |
| 5,6 | 3 x 1.02 d (6.89) | 18,25 | 3,6 |  |
|  |  |  |  |  |
| **N-methyl-valine 6** |  |  |  |  |
| 1,7 |  | 167,57 |  |  |
| 2,7 | 3.68 d (5.34) | 67,9 | 3,7 | 1.7, N-methyl |
| 3,7 | 2.18 m | 31,35 | 2.7, 4.7, 5.7 | 1,7 |
| 4,7 | 3 x 1.05 d (6.98) | 18,25 | 3,7 |  |
| 5,7 | 3 x 1.02 d (6.89) | 18,25 | 3,7 |  |
| 6,7 | 3 x 2.65 s | 32,99 |  | 2,7 |

pos. indicates position, multi indicates multiple.

**
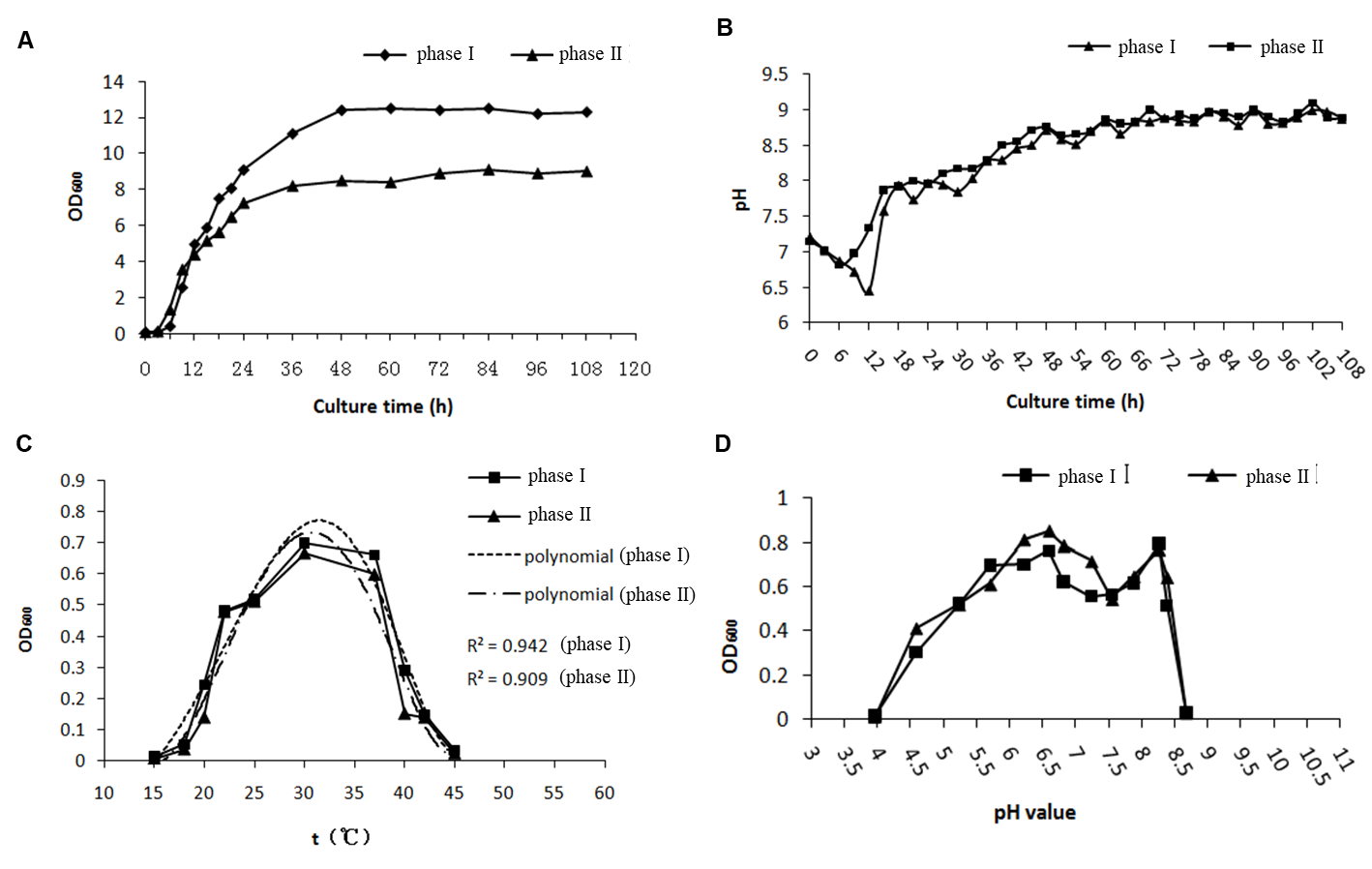
**

**FIGURE S1.** Growth characteristics of strain HN_xs01 in LB broth. (A) Growth curves of phases I and II of HN_xs01 in LB broth. (B) Changes in broth pH during culturing of strain HN_xs01 phases. (C) The optimum growth temperature for different phases of strain HN_xs01. (D) The optimum growth pH for different phases of strain HN_xs01. Phase I indicates the primary form, and phase II indicates the secondary form.

**
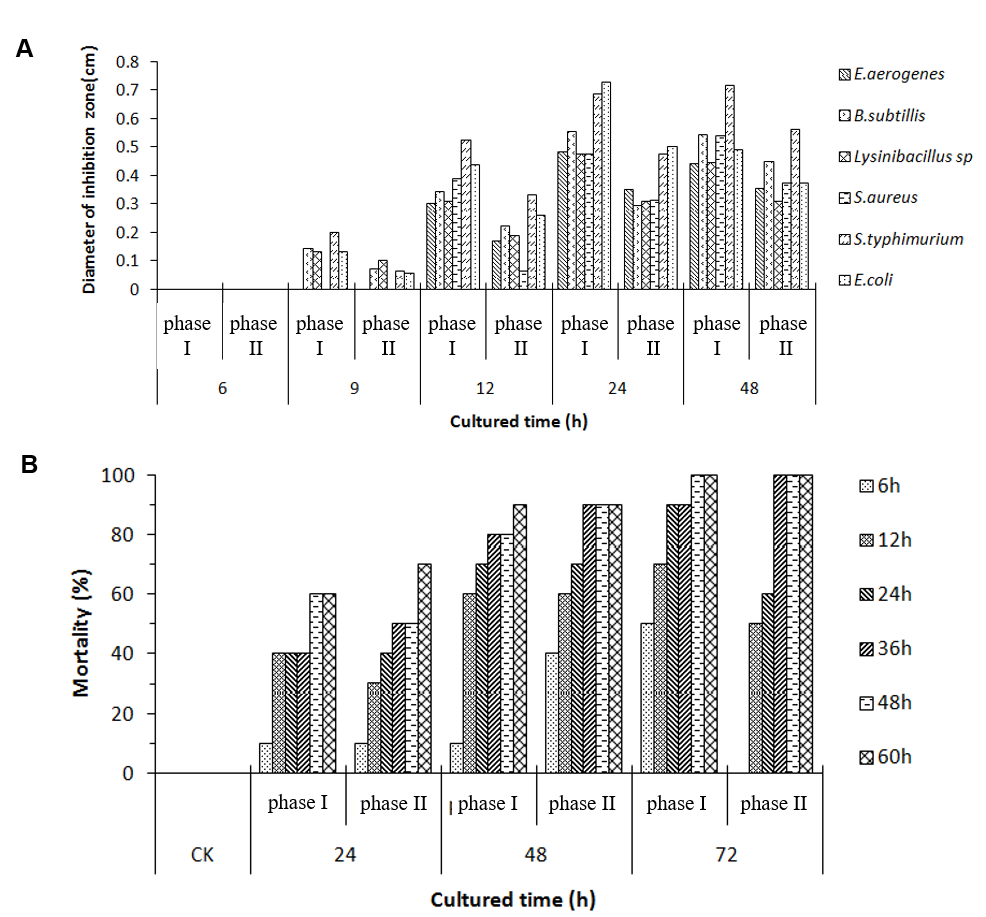
**

**FIGURE S2.** Antimicrobial and insecticidal activities of the different forms of strain HN_xs01. (A) Antimicrobial activity of 6 h, 9 h, 12 h, 24 h, and 48 h cultures of HN_xs01. The 9 h culture of HN_xs01 showed some inhibitory activity against the tested bacteria, and bacteriostatic activity peaked with the 24 h culture. The antibacterial activities were most effective against *S. typhimurium* and *E. coli,* reaching a maximum inhibition zone of 7 mm (diameter). The primary form showed better bacteriostatic efficacy than the secondary form. (B) Insecticidal activity of 24 h, 48 h, and 72 h culture of strain HN_xs01, respectively. The 72-h culture of HN_xs01 had the strongest insecticidal toxicity; mortality of *H. armigera* was as high as 50% by 6 h after injection and reached 100% after 48 h. There was no significant difference between the primary form and secondary form. No deaths were detected in the control group (CK).

**
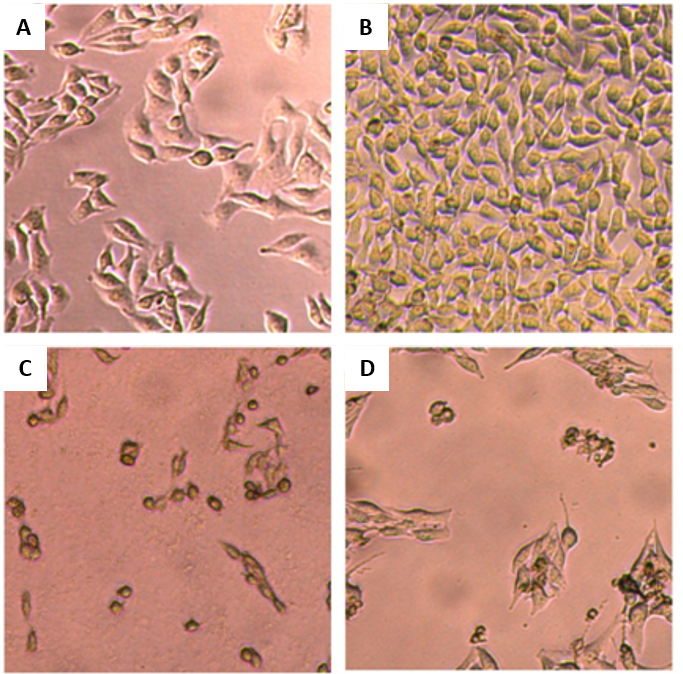
**

**FIGURE S3.** Cytotoxic activity of 72-h culture of strain HN_xs01 *in vitro*. (A) B16 cells before treatment. (B) B16 cells treated with sterile LB. (C) B16 cells treated with 72 h culture supernatant of the primary form. (D) B16 cells treated with 72 h culture supernatant of the secondary form. Equal volumes of LB (B) or culture (C, D) were used.


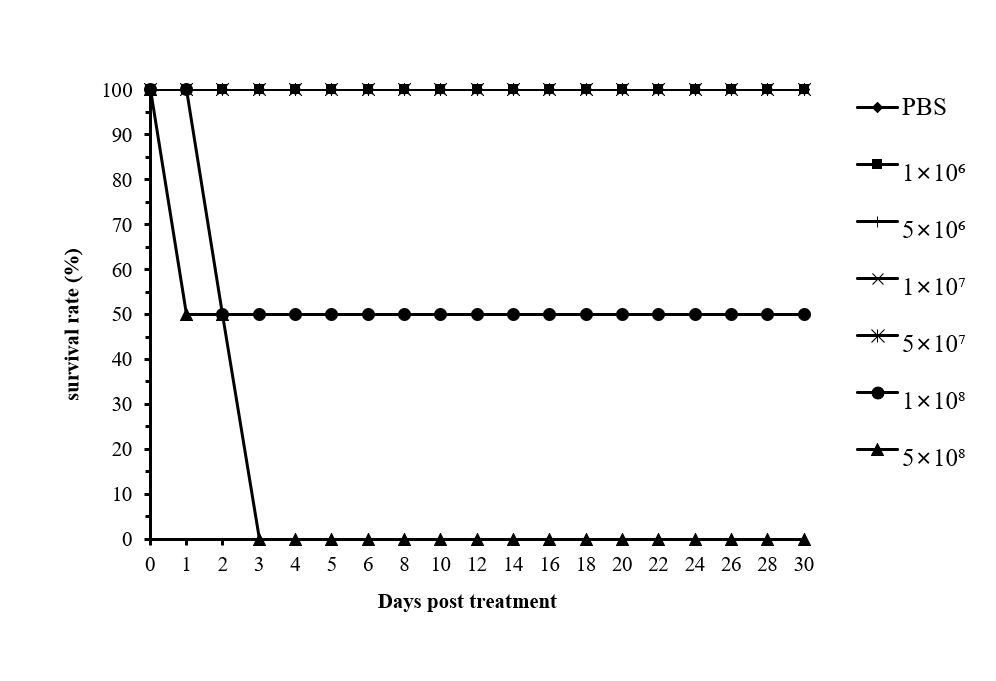


**FIGURE S4.** Tolerance of C57BL/C mice for HN_xs01 administered by intravenous injection. C57BL/C mice bearing B16 tumors were injected intravenously with different amounts of strains HN_xs01, and the control group (CK) was that C57BL/C mice bearing B16 tumors injected with the equivalent volume of PBS. The survival rate was determined over 10 days post-injection.

**FIGURE S5.** Structures of **1**, **2**, and **3**.

**
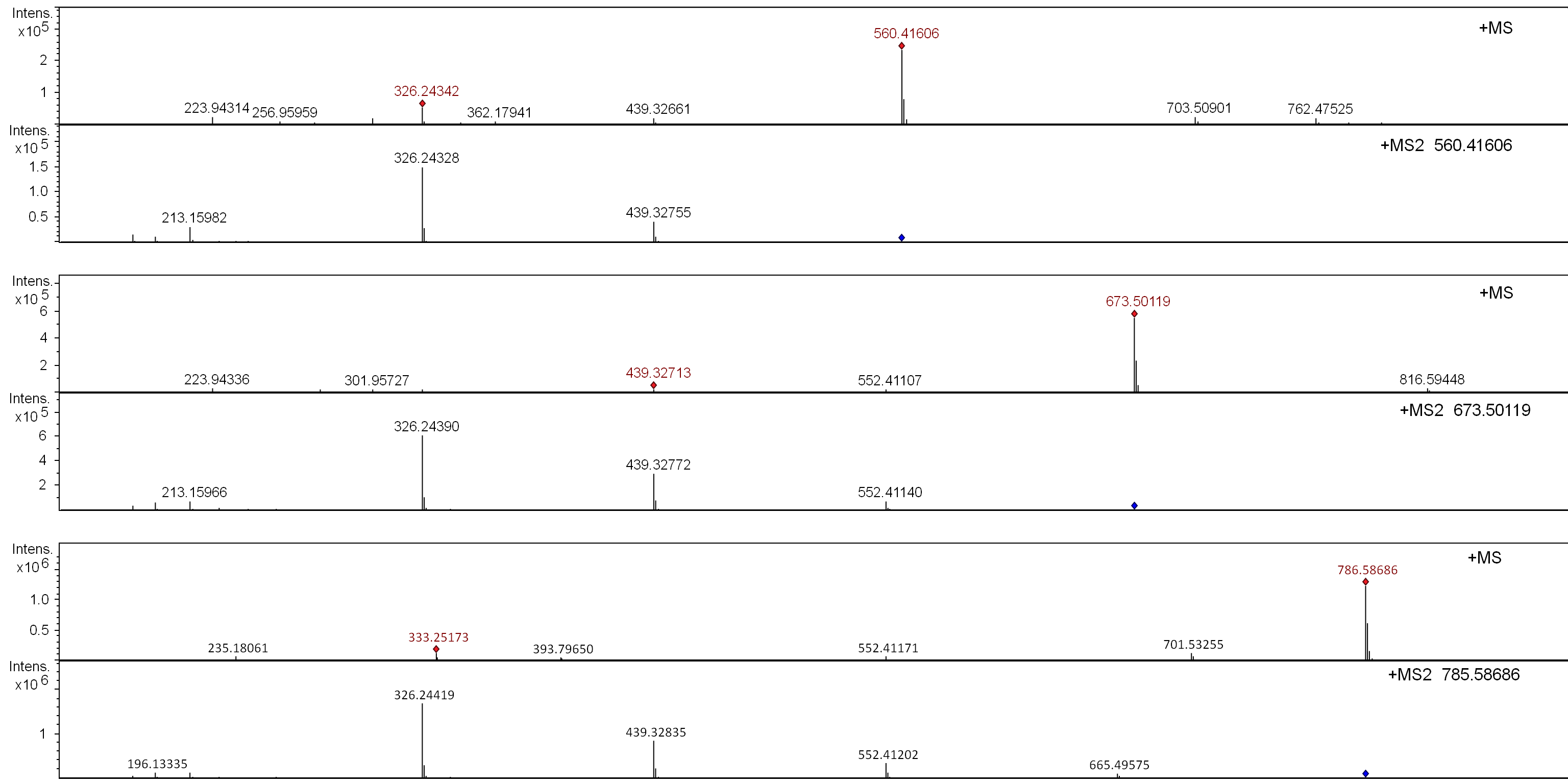
**

**FIGURE S6** High-resolution ESI-MS and MSMS of three linear rhabdopeptides.

**FIGURE S7.** ^1^H-NMR spectra of **1** in MeOD-*d_4_* at 700 MHz.

**FIGURE S8.** HSQC-NMR spectra of **1** in MeOD-*d_4_* at 700 MHz.

**FIGURE S9.** HMBC-NMR spectra of **1** in MeOD-*d_4_* at 700 MHz.

**FIGURE S10.** COSY-NMR spectra of **1** in MeOD-*d_4_* at 700 MHz.

**FIGURE S11.** ^1^H-NMR spectra of **2** in MeOD-*d_4_* at 700 MHz.

**

**FIGURE S12.** HSQC-NMR spectra of **2** in MeOD-*d_4_* at 700 MHz.

**

**FIGURE S13.** HMBC-NMR spectra of **2** in MeOD-*d_4_* at 700 MHz.

**FIGURE S14.** COSY-NMR spectra of **2** in MeOD-*d_4_* at 700 MHz.

**FIGURE S15.** ^1^H-NMR spectra of **3** in MeOD-*d_4_* at 700 MHz.

**FIGURE S16.** HSQC-NMR spectra of **3** in MeOD-*d_4_* at 700 MHz.

**FIGURE S17.** HMBC-NMR spectra of **3** in MeOD-*d_4_* at 700 MHz.

**FIGURE S18.** COSY-NMR spectra of **3** in MeOD-*d_4_* at 700 MHz.
